# *Cichorium intybus*: Traditional Uses, Phytochemistry, Pharmacology, and Toxicology

**DOI:** 10.1155/2013/579319

**Published:** 2013-11-26

**Authors:** Renée A. Street, Jasmeen Sidana, Gerhard Prinsloo

**Affiliations:** ^1^Medical Research Council, HIV Prevention Research Unit, Westville, Durban 4041, South Africa; ^2^School of Pharmaceutical Sciences, Lovely Professional University, Jalandhar-Delhi G.T. Road (NH-1), Phagwara, Punjab 144411, India; ^3^Department of Agriculture and Animal health, University of South Africa (UNISA), Florida Campus, Florida 1710, South Africa

## Abstract

The genus *Cichorium* (Asteraceae) is made up of six species with major geographical presence in Europe and Asia. *Cichorium intybus*, commonly known as chicory, is well known as a coffee substitute but is also widely used medicinally to treat various ailments ranging from wounds to diabetes. Although this plant has a rich history of use in folklore, many of its constituents have not been explored for their pharmacological potential. Toxicological data on *C. intybus* is currently limited. This review focuses on the economic and culturally important medicinal uses of *C. intybus*. Traditional uses, scientific validation, and phytochemical composition are discussed in detail.

## 1. Introduction

The genus *Cichorium* (Asteraceae) consists of six species with major distribution areas in Europe and Asia [1]. In several Asteraceae, inulin, a **β**-2,1 linked fructose polymer with a terminal glucose residue, functions as a reserve carbohydrate in stems, tubers, and taproots [2]. *Cichorium intybus* L., commonly known as chicory, is an erect fairly woody perennial herb, around 1 m in height with a fleshy taproot of up to 75 cm in length and large basal leaves [1, 3]. Historically, chicory was grown by the ancient Egyptians as a medicinal plant, coffee substitute, and vegetable crop and was occasionally used for animal forage. In the 1970s, it was discovered that the root of* C. intybus *contained up to 40% inulin, which has a negligible impact on blood sugar and thus is suitable for diabetics [4]. To date, *C. intybus* is grown for the production of inulin on an industrial scale [2]. The name of the plant is derived from Greek and Latin. *Cichorium *means *field* and *intybus* is partly derived from the Greek “to cut”, because of the leaves, and partly from the Latin *tubus* to indicate the hollow stem [5].

Chicory is a hardy plant and can endure extreme temperatures during both vegetative and reproductive growth stages [1]. When broken, all plant parts exudate a milky latex [3]. *Cichorium intybus* is cultivated for numerous applications and can be divided into four main varieties or cultigroups according to their use [6]: (1) “industrial” or “root” chicory, predominantly cultivated in northwestern Europe, India, South Africa, and Chile, produces the taproot as a coffee substitute or for inulin extraction; (2) “Brussels” or “witloof” chicory is commonly cultivated around Europe as industrial chicory for etiolated buds (chicons) by forcing; (3) “leaf” chicory is used as fresh or cooked vegetables; and (4) “forage” chicory, initially derived from wild chicory commonly found along roadsides and waste areas, has been used since the mid-1970s to intensify herbage obtainability in perennial pastures for livestock.

*Cichorium intybus *is a medicinally important plant in Eurasia and in parts of Africa. Despite its long tradition of use, the plant is not described in the European Pharmacopoeia or in any official Pharmacopoeia of a European Union member state [5]. However, due to its prevalent distribution, different parts of the plant have been used in traditional medicines globally [7]. Important phytochemicals are distributed throughout the plant, but the main contents are present in the root [1]. This review focuses on the economic and culturally important medicinal uses of *C. intybus. *Traditional uses, scientific validation, and phytochemical composition are discussed in detail.

## 2. Traditional Uses

Medicinal plants have been used for centuries and numerous cultures still rely on indigenous medicinal plants to meet their primary health care needs. It is likely that the insightful knowledge of plant-based remedies in traditional cultures advanced through trial and error and that the most important cures were carefully passed from one generation to another [8]. Historically, chicory was grown by the ancient Egyptians as a medicinal plant [9] and it has had a long history of therapeutic use both in areas where it is indigenous and in areas where it has been introduced. The various common or local names describing this plant may be ascribed to the widespread use by different folkloric groups. 

Different preparations of this plant are employed to treat various symptoms and ailments (Table 1). The juice is said to be a folk remedy for cancer of the uterus and for tumors [4]. In South Africa, although it is considered a widespread weed, leaves, stems, and roots are made into a tea for jaundice and chicory syrup is used as a tonic and purifying medicine for infants [3]. In Turkey, an ointment is made from the leaves for wound healing [10]. Decoction refers to a preparation that is made by adding cold water to the plant material which is then boiled and allowed to simmer for 5–10 min after which it is strained [8]. Chicory decoctions are traditionally made from individual plant parts and/or from the plant as a whole (Table 1).

According to the European monograph, traditional use of chicory roots includes the relief of symptoms related to mild digestive disorders (such as feeling of abdominal fullness, flatulence, and slow digestion) and temporary loss of appetite [11]. Prior to the wars in Afghanistan, folkloric reports described the use of aqueous root extracts as a light-sensitive plant remedy for malaria. This indigenous knowledge has since been confirmed and the antimalarial compounds of *C. intybus* roots have been identified as the light-sensitive sesquiterpene lactones lactucin and lactucopicrin [12]. The flowers of the chicory plant (*Cichorii flos*) are used as a herbal treatment of everyday ailments such as a tonic and appetite stimulant and as a treatment of gallstones, gastroenteritis, sinus problems, cuts, and bruises [4]. In Italy, the whorls are made into a decoction and used as a depurative [13]. Chicory seeds are one of the main ingredients of *Jigrine,* a commercial product of India used for the treatment of various diseases of the liver [14]. Other plant parts are also used for liver disorders, namely, aerial parts in Bosnia and Herzegovina [15] and roots in Serbia and India [16, 17].

## 3. Chemical Constituents

Chicoric acid has been identified as the major compound in methanolic extracts of chicory (Table 2) [18]. Aliphatic compounds and their derivatives comprise the main fraction while terpenoids comprise minor constituents of the plant. The flowers of chicory contain saccharides, methoxycoumarin cichorine, flavonoids, essential oils [4], and anthocyanins contributing to the blue colour of the perianth [19]. Table 2 provides a summary of the compounds isolated and identified from chicory. Octane, *n*-nonadecane, pentadecanone, hexadecane, and a tentatively identified compound have been found as principal volatile components [4]. A list of volatile compounds is given in Table 3.

## 4. Pharmacological Activities 

*Cichorium intybus* presents a little investigated plant in terms of phytochemistry and pharmacology. Over 100 individual compounds have been isolated and identified from this plant (Table 2), the majority of which are from the roots. Most of the pharmacological studies on this plant document the testing of aqueous and/or alcoholic extracts only. Apart from the pharmacologically important activities, the use of *C. intybus* (hairy root cultures) has also been implicated in the phytoremediation of DDT [20].

### 4.1. Antimicrobial Activity

The antibacterial activity of the organic acid-rich extract of fresh red chicory (*C. intybus* var. sylvestre) was tested against periodontopathic bacteria including *Streptococcus mutans*, *Actinomyces naeslundii,* and *Prevotella intermedia*. The compounds identified from the active extract include oxalic acid, succinic acid, quinic acid, and shikimic acid. All of the organic acids were found to decrease biofilm formation and adhesion of bacteria to the cells, with different levels of efficacy. These compounds also induced biofilm disruption and detachment of dead cells for the cultured substratum [21]. In other reports on the antimicrobial activity of *C. intybus*, the crude aqueous and organic seed extracts were found to be active against four pathogenic microorganisms, namely, *Staphylococcus aureus*, *Pseudomonas aeruginosa*, *Escherichia coli,* and *Candida albicans,* and root extracts had pronounced effects on *Bacillus subtilis*, *S. aureus*, *Salmonella typhi*, *Micrococcus luteus*, and *E. coli *[22, 23]. The leaf extract of *C. intybus* also showed a moderate activity against multidrug resistant *S. typhi *[24]. Guaianolides-rich root extracts of *C. intybus* have shown antifungal properties against anthropophilic fungi *Trichophyton tonsurans*, *T. rubrum,* and *T. violaceum *[25]. A sesquiterpenoid phytoalexin cichoralexin isolated from chicory exhibited potent antifungal activity against *Pseudomonas cichorii *[26].

### 4.2. Anthelmintic Activity

Several studies have been conducted on grazing animals to determine the anthelmintic potential of secondary metabolites present in *C. intybus*. Grossly, it has been concluded that the animals grazing on chicory have a higher performance index and lower incidence of gastrointestinal nematode infestations. In the majority of the experiments, the condensed tannins and sesquiterpene lactones were responsible for anthelmintic activity [27]. Anthelmintic activity of chicory has also been noticed in the case of lambs wherein the total number of abomasal helminths was found to be lesser in the lambs grazing on this plant [28]. The condensed tannin and sesquiterpene-rich extracts of *C. intybus* were evaluated for their efficacy against the larvae of deer lungworm, *Dictyocaulus viviparous* and other gastrointestinal nematode larvae using a larval migration inhibition assay. A dose-dependent decrease in the larval motility was observed in both lungworm and gastrointestinal nematodes [29]. The sesquiterpene lactone-rich extracts of *C. intybus* were also found to inhibit egg hatching of *Haemonchus contortus *[30].

### 4.3. Antimalarial Activity

The infusion of fresh roots of *C. intybus* has a history of use as a remedy for malarial fevers in some parts of Afghanistan. The bitter compounds in the plant, namely, lactucin, lactucopicrin, and the guaianolide sesquiterpenes, isolated from aqueous root extracts of chicory were concluded to be the antimalarial components of the plant. Lactucin and lactucopicrin completely inhibited the HB3 clone of strain Honduras-1 of *Plasmodium falciparum* at concentrations of 10 and 50 *μ*g/mL, respectively [12, 31].

### 4.4. Hepatoprotective Activity

The folkloric use of *C. intybus* as a hepatoprotectant has been well documented. It is one of the herbal components of Liv-52, a traditional Indian tonic used widely for hepatoprotection. In a randomized, double-blind clinical trial conducted on cirrhotic patients, Liv-52 medication reduced the serum levels of hepatic enzymes, namely, alanine aminotransferase and aspartate aminotransferase. It also reduced the Child-Pugh scores and ascites significantly [32]. Another polyherbal formulation,* Jigrine*, contains the leaves of *C. intybus* as one of its 14 constituents. *Jigrine* was evaluated for its hepatoprotective activity against galactosamine-induced hepatopathy in rats. The pretreatment of male Wistar-albino rats with *jigrine* significantly reduced the levels of aspartate transaminase, alanine transaminase, and urea and increased the levels of blood and tissue glutathione. Histopathological examination of the liver revealed that *jigrine* pretreatment prevented galactosamine toxicity and caused a marked decrease in inflamed cells [33].

The aqueous-methanolic extract of the seeds of *C*. *intybus* has been investigated for the hepatoprotective activity against acetaminophen and carbon tetrachloride-induced liver damage in mice. It was found to decrease both the death rate and the serum levels of alkaline phosphatase, glutamyl oxaloacetate transaminase, and glutamyl pyruvate transaminase [34]. In analogous studies, the antihepatotoxic activity of the alcoholic extract of the seeds and aqueous extracts of the roots and root callus of *C*. *intybus* was estimated. The oral administration of these extracts in albino rats led to a marked decrease in the levels of hepatic enzymes. Also, histopathological examination of the liver showed no fat accumulation or necrosis after the treatment [14, 35]. Similar studies have established the hepatoprotective effect of esculetin, a phenolic compound, and cichotyboside, a guaianolide sesquiterpene glycoside reported from *C. intybus *[36, 37].

The carbon tetrachloride and paracetamol-induced liver toxicities were also found to be counteracted by intraperitoneal administration of crude extracts and fractions of *C. intybus*. The methanol- and water-soluble fractions exhibited marked reductions in serum glutamyl pyruvate transaminase, serum glutamyl oxaloacetate transaminase, alkaline phosphatase, and total bilirubin levels. In the same study, toxicity was induced in rat hepatocytes by incubation with galactosamine and thioacetamide [38].

The phenolic acid-rich seed extract of *C. intybus* was evaluated for its efficacy against hepatic steatosis *in vitro* and *in vivo*. The *in vitro* model of hepatic steatosis was created by incubation of the HepG2 cells with oleic acid leading to intracellular accumulation of fat. The seed extract was effective in decreasing the deposited fat from the cells in case of administration after the initial fat deposition (i.e., nonsimultaneous administration with oleic acid). However, in case of simultaneous administration of seed extract and oleic acid, the extract could not protect the cells from steatosis except at very high doses. The extract also led to the increased release of glycerol (an indicator of triglyceride degradation) in steatotic cells. In case of nonsimultaneous administration, the extract was found to upregulate the expression of SREBP-1c and PPAR-**α** genes leading to restoration of normal levels of corresponding proteins. In the *in vivo* model of hepatic steatosis, namely, diabetic rats, treatment with seed extract resulted in significant decrease in fat accumulation and fibrosis [39].The hepatoprotective activity of *C. intybus* has been correlated to its ability to inhibit the free radical mediated damage. A fraction prepared from the ethanolic extract of the leaves was assessed for preventive action on the free radical mediated damage to the deoxyribose sugar of the DNA (obtained from calf thymus). A dose-dependent decrease in the DNA damage was observed in the present assay [40].

### 4.5. Antidiabetic Activity

Chicory has reported antidiabetic activity [17, 41]. Based on the traditional use of *C. intybus* in diabetes mellitus, the hypoglycemic and hypolipidemic properties of the ethanol extract of the whole plant were investigated. Diabetes was induced by intraperitoneal administration of streptozotocin in male Sprague-Dawley rats. The ethanol extract, at a dose of 125 mg/Kg body weight, significantly attenuated the serum glucose levels in the oral glucose tolerance test. A marked decrease in the serum triglycerides and cholesterol was also observed in the extract-treated rats. Hepatic glucose-6-phosphatase activity was found to be reduced in extract-treated diabetic rats as compared to untreated diabetic rats [17]. The antidiabetic effect of the aqueous seed extract of *C. intybus* has also been investigated. Early-stage and late-stage diabetes were differently induced in male Wistar albino rats by streptozotocin-niacinamide and streptozotocin alone, respectively. The treatment with chicory extract prevented weight loss in both early-stage and late-stage diabetic rats. Chicory-treated diabetic animals resisted excessive increase in fasting blood sugar (assessed by glucose tolerance test). Grossly, normalization of blood parameters, namely, alanine aminotransferase, triacylglycerol, total cholesterol, and glycosylated heamoglobin, was seen in these animals. In early-stage diabetic rats, chicory treatment led to the increase in insulin levels pointing toward the insulin-sensitizing action of chicory [42].

Feeding the diabetic Wistar rats with *C. intybus* leaf powder led to a decrease in blood glucose levels to near normal value. *C. intybus *administration also decreased the malondialdehyde (formed by thiobarbituric acid) levels and increased glutathione content. Anticholinesterase activity was restored to near normal, brain lipopolysaccharide decreased, and catalase activity increased [43]. Caffeic acid and chlorogenic acid have been described as potential antidiabetic agents by increasing glucose uptake in muscle cells. Both compounds were also able to stimulate insulin secretion from an insulin-secreting cell line and islets of Langerhans. Another compound, chicoric acid, is also a new potential antidiabetic agent exhibiting both insulin-sensitizing and insulin-secreting properties [44].

### 4.6. Gastroprotective Activity

*C. intybus* has been used in Turkish folklore for its antiulcerogenic potency. The aqueous decoction of *C. intybus* roots was orally administered to Sprague-Dawley rats 15 minutes before the induction of ulcerogenesis by ethanol. More than 95% inhibition of ulcerogenesis was observed in the test group [45].

### 4.7. Anti-Inflammatory Activity

The inhibition of TNF-**α** mediated cyclooxygenase (COX) induction by chicory root extracts was investigated in the human colon carcinoma (HT 29) cell line. The ethyl acetate extract inhibited the production of prostaglandin E_2_ (PGE_2_) in a dose-dependent manner. TNF-**α** mediated induction of COX-2 expression was also suppressed by the chicory extract [46].

### 4.8. Analgesic Activity

Lactucin, lactucopicrin, and 11**β**, 13-dihydrolactucin exhibited analgesic action in mice in hot plate and tail-flick tests. In the hot plate test, all three compounds exerted an analgesic effect, with lactucopicrin being the most potent compound. In the tail-flick test, the antinociceptive effects of all the tested compounds (30 mg/kg dose) were comparable to that of ibuprofen (60 mg/kg dose). Lactucin and lactucopicrin were also established to have some sedative action as evident from the decreased spontaneous locomotor activity in mice [47].

### 4.9. Antioxidant Activity

The DPPH radical scavenging activity of a polyphenols-rich fraction of *C. intybus* has been investigated [48]. The anti- and prooxidant activities of *Cichorium* species were studied in chemical as well as biological systems. In the case of chemical systems, the antioxidant activity of water-soluble compounds in *C. intybus* var. *silvestre* was established in the coupled model of linoleic acid and **β**-carotene. A pro-oxidant activity of some of the chemical components was recorded initially which notably diminished with time and/or thermal treatment. Thereafter, the antioxidant activity of the raw juice and its fractions persisted. The molecular weight ranges of the antioxidant fractions of raw juice were also identified based on dialysis [49]. Two varieties of chicory, namely, *C. intybus* var. *silvestre* and *C. intybus* var. *foliosum*, have been investigated for their antioxidant (antiradical) activities in two distinct biological systems. The lipid peroxidation assay has been carried out on microsome membranes of rat hepatocytes after the induction of oxidative damage by carbon tetrachloride. The antiradical activity was expressed as the protective activity against lipid peroxidation and calculated as the percentage decrease in hydroperoxide degradation products. The second biological system used was the cultures of *S. aureus* after treatment with cumene hydroperoxide. The percentage increase of growth of bacteria was noted after the treatment with juices of chicory varieties. In both systems, the juices of chicory varieties showed strong antiradical activities [21, 49].

Red chicory (*C. intybus* var. *silvestre*) was studied for its polyphenol content and the antioxidant activity was evaluated by using the synthetic 2,2-diphenyl-1-(2,4,6-trinitrophenyl) hydrazyl radical and three model reactions catalyzed by pertinent enzymatic sources of reactive oxygen species, namely, xanthine oxidase, myeloperoxidase, and diaphorase. Total phenolics were significantly correlated with the antioxidant activity evaluated with both the synthetic radical and the enzyme-catalyzed reactions. On a molar basis, red chicory phenolics were as efficient as Trolox (reference compound) in scavenging the synthetic radical [50]. The aqueous-alcoholic extracts of the aerial parts of *C. intybus* also inhibited xanthine oxidase enzyme dose dependently [51]. In another study, along with DPPH radical scavenging activity, *C. intybus* also exhibited inhibition of hydrogen peroxide and chelation of ferrous ion [52].

### 4.10. Tumor-Inhibitory Activity

The crude ethanolic extract of *C. intybus* roots caused a significant inhibition of Ehrlich tumor carcinoma in mice. A 70% increase in the life span was observed with a 500 mg/kg/day intraperitoneal dose of the tested extract [53]. The aqueous-alcoholic macerate of the leaves of *C. intybus *also exerted an antiproliferative effect on amelanotic melanoma C32 cell lines [54]. Magnolialide, a 1**β**-hydroxyeudesmanolide isolated from the roots of *C. intybus*, inhibited several tumor cell lines and induced the differentiation of human leukemia HL-60 and U-937 cells to monocyte or macrophage-like cells [55].

### 4.11. Antiallergic Activity

The aqueous extract of *C. intybus* inhibited the mast cell-mediated immediate allergic reactions *in vitro* as well as *in vivo*. This extract restrained the systemic anaphylactic reaction in mice in a dose-dependent manner. It also significantly inhibited passive cutaneous anaphylactic reaction caused by anti-dinitrophenyl IgE in rats. Other markers of allergic reaction, namely, plasma histamine levels and histamine release from rat peritoneal mast cells, decreased significantly whereas the levels of cAMP increased after the treatment with *C. intybus* extract [56].

### 4.12. Other Pharmacologically Important Activities

The ethanol extract of the roots of *C. intybus* is reported to prevent the immunotoxic effects of ethanol in ICR mice. It was noted that body weight gains were markedly decreased in mice administered with ethanol. However, the body weight was not affected when ethanol was coadministered with the ethanol extract of *C. intybus*. Similarly, the weights of liver and spleen were not affected when ethanol extract was given along with ethanol. A considerable restoration in the other markers of immunity, namely, hemagglutination titer, plaque forming cells of spleen, secondary IgG antibody production, delayed-type hypersensitivity reaction (in response to subcutaneous administration of sheep red-blood cells to paw), phagocytic activity, number of circulating leucocytes, NK cell activity, cell proliferation, and production of interferon-*γ*, was registered [57]. The immunoactive potential of an aqueous-alcoholic extract of the roots was established by a mitogen proliferation assay and mixed lymphocyte reaction (MLR). The extract showed an inhibitory effect on lymphocyte proliferation in the presence of phytohemagglutinin and a stimulatory effect on MLR [58].

Chicoric acid has shown vasorelaxant activity against nor-epinephrine-induced contractions in isolated rat aorta strips [59]. A pronounced anticholinesterase activity of the dichloromethane extract of *C. intybus* roots was seen in the enzyme assay with Ellman's reagent. Two sesquiterpene lactones, namely, 8-deoxylactucin and lactucopicrin, also exhibited a dose-dependent inhibition of anticholinesterase [60]. The methanolic extract displays wound healing effect and **β**-sitosterol was determined as the active compound responsible for the activity, possibly due to its significant anti-inflammatory and antioxidant effects, as well as hyaluronidase and collagenase inhibition [7].

## 5. Toxicological Studies

Although *C. intybus* has a long history of human use, the high levels of secondary metabolites have shown potential toxicological effects. To evaluate the safety of the root extract of *C. intybus*, Ames test and subchronic toxicity assessment were conducted. The sesquiterpene-rich extract was evaluated for potential mutagenic properties (Ames test) using *Salmonella typhimurium* strains TA97a, TA98, TA100, and TA1535 and *Escherichia coli* strain WP2 *uvrA*. Though cytotoxicity was observed at high extract doses in some strains, mutagenicity was not noted. A 28-day (subchronic) oral toxicity study, conducted in CRL:CD (SD) IGS BR rats, concluded that there was no extract-related mortality or any other signs of toxicological significance [61]. The toxicity evaluation of *C. intybus* extracts has also been done by *Vibrio fischeri* bioluminescence inhibition test (Microtox acute toxicity test). This bacterial test measures the decrease in light emission from the marine luminescent bacteria *V. fischeri *when exposed to organic extracts. The tested extracts showed less than 20% inhibition of bioluminescence and hence were concluded to be safe for human use [54].

## 6. Clinical Trials

Two clinical studies on chicory roots are reported in the literature, both of which are pilot studies and are therefore considered to be insufficient to support a well-established use indication for chicory root [5]. The first study, a phase 1, placebo-controlled, double-blind, dose-escalating trial, was conducted to determine the safety and tolerability of a proprietary bioactive extract of chicory root in patients with osteoarthritis (OA) [62]. In general, the treatment was well tolerated. Only one patient who was treated with the highest dose of chicory had to discontinue treatment due to an adverse event. The results of the pilot study suggested that a proprietary bioactive extract of chicory root has a potential role in the management of OA and merits further investigation. The second pilot study was conducted to assess whether chicory coffee consumption might confer cardiovascular benefits; thus, a clinical intervention was performed with 27 healthy volunteers, who consumed 300 mL chicory coffee daily for one week [63]. Depending on the inducer used for the aggregation test, the dietary intervention showed variable effects on platelet aggregation. Whole blood and plasma viscosity were both significantly reduced, along with serum MIF levels, after a week of chicory coffee intake. It was concluded that the full spectrum of the effects was unlikely to be attributed to a single phytochemical; nevertheless, the phenolics (including caffeic acid) are expected to play a substantial role. The study offered an encouraging starting-point to describe the antithrombotic and anti-inflammatory effects of phenolic compounds found in chicory coffee.

In the European Union, there is currently only one registered/authorized herbal medicinal product containing *C. intybus *as single ingredient whilst there are several combination products on the market [5]. The efficacy of herbal medicine Liv-52 consisting of *Mandur bhasma, Tamarix gallica,* and herbal extracts of *Capparis spinosa, C. intybus, Solanum nigrum, Terminalia arjuna,* and *Achillea millefolium* on liver cirrhosis outcomes was compared with the placebo for 6 months in 36 cirrhotic patients. The study concluded that Liv-52 possessed a hepatoprotective effect in cirrhotic patients. This protective effect of Liv-52 can be attributed to the diuretic, anti-inflammatory, anti-oxidative, and immunomodulating properties of the component herbs [32].

## 7. Cultivation and Sustainable Use

Greeks and Romans began to grow chicory as a vegetable crop 4000 years ago [9]. Since the discovery in the 1970s that chicory root contains up to 40% inulin (polysaccharide), new strains have been created, with inulin content comparable to that of sugar beet [4]. It is a common vegetable in several Western European countries. It is typically grown in a biennial cycle, with a tuberised root produced during the vegetative growth phase [64]. During the first field year, the vegetative growth phase is characterized by the production of a fleshy taproot. The second field year is the generative phase in which the flowering stem is formed and seeds are produced. To produce the eatable leafy vegetable called a chicon, roots are harvested at the end of the first growing period when an appropriate stage of maturity is reached [65]. The application of inulin in the food industry was restricted to the production of coffee substitutes. It was later discovered that inulin could act as a substitute for sugar or fat due to its low caloric value. The most stable form for the commercialization of inulin is the powdered extract for its greater facility of manipulation, transport, storage, and consumption [66].

Chicory is especially attractive as a cash crop since it can reach more than 62 t ha^−1^ under favourable conditions. Inulin content can reach on average 15% of root fresh weight and a yield of 8 t ha^−1^ of inulin is achievable [67]. The USA imports more than 2.3 million kilograms of chicons and 1.9 million kilograms of roasted chicory roots for coffee according to 2002 US Department of Commerce tariff and trade data [61]. Numerous studies have focused on different cultivation aspects of chicory. Chicory is considered one of the most important sources of inulin since it has a high root yield potential and also a high root sugar content [68]. A high root yield, a high inulin content, and especially long inulin chains are preferred [69]. Short chain inulin is used for the production of fructose syrup used in sweetening of cold drinks whereas long chain inulin is used as fat replacer and foam stabilizer in food products and also in the production of carboxymethyl inulin [2]. The effect of fertilizers on the growth, development, and yield of chicory has been well studied. In general, increase of nitrogen (N) increases the growth and ultimately the yield, although a high application of N has a negative effect on especially some of the amino acids. Increased N application at levels of 200 kg N ha^−1^ leads to a decrease in amino acids such as threonine and valine with a pronounced decreased effect on methionine. A level of 100 kg N ha^−1^ is preferred for enhanced quality [70]. In terms of phosphorous (P), it has been established that chicory has at least two inherent patterns of response to low or zero P conditions. One pattern is the classical increase in the length of the smallest diameter roots in response to P deficient conditions. The second pattern is a significant decrease in root tissue density under low P conditions [71]. It has also been investigated as a suitable catch crop since it has the ability to withdraw N, especially nitrate, from the soil, thereby reducing potential leaching. Additionally, it can withstand competition, has a slow juvenile development and vigorous growth after harvest of the main crop, is frost- and winter-hardy, has a well-developed root system, and does not transmit pathogens or pests to other crops [72]. Canopy closure in chicory is advantageous and critical for yield and can be achieved by a larger supply of assimilates to the shoot [73].

Chicory is greatly influenced by the pH of the soil as this affects the availability of nutrients in the soil to the plant. A study conducted by Anguissola Scotti el al. [74] on two soils of pH, 5.7 and 7.0 indicated that the fly-ash or metal availability to the plant was significantly different at the different pH levels. At a low pH a decrease of Zn, Cu, Cd, and Ni was observed and for neutral soils the added metals are more available to plants than those naturally occurring in soils [75]. Chicory is a cold-requiring long-day plant [72]. In a study by Amaducci and Pritoni [68], it was shown that retarding harvest time significantly affects the content and concentration of inulin. Rainfall has proven to be another important factor since the roots contain a higher water content, thereby affecting the concentration of inulin in the roots. The same results have been obtained in a study by Baert [69] where an early sowing date and harvesting time increased the root yield, total sugar content, and inulin chain length. The increase in yield with an earlier sowing date was up to 30% higher with an increase of 10% with an earlier harvest date. The content of free fructose and sucrose increased and the content of free glucose and inulin decreased with a later harvest time [75].

Even though chicory is a cold-requiring plant, cold storage after harvest can cause a strong decrease in free glucose, an increase in free fructose and sucrose, and hydrolysis of inulin [76]. This has also been observed by Ernst et al. [77] where an onset of cooler temperatures and especially colder temperatures during storage resulted in an increase in sucrose and fructose content. It was found that sucrose increased in the roots of chicory about threefold and fructose increased about tenfold within the first few weeks of cold storage after harvest [77]. The effect of storage at reduced temperature has also been studied on the levels of sesquiterpene lactones. Storage at 2°C and 10°C for up to 13 days had no effect on the level of lactucin-like sesquiterpene lactones in the chicons and after 7 days of storage a slight increase of lactucopicrin content was observed [78]. Several types of discoloration, leaf edge damage, and extensive growth of the internal core can occur in the heads of chicory during postharvest storage, which considerably reduces their market value. An atmospheric composition of 10% O_2_ and 10% CO_2_ in combination with a storage temperature of 5°C was found optimal [64]. Low temperature in the field also hastens and enhances bolting and flowering [79, 80]. The type and cultivar have been identified in numerous studies to be the determining factor during the stages of growth [81]. Suhonen [82] found the highest numbers of bolters in those being planted last, for which the mean temperatures during early growth were the highest.

During the postharvest period, the major polyamines present are putrescine, especially in the oldest leaves, although spermidine is present in considerable amounts, showing a tendency to decrease with the increasing physiological age of the leaves. Free sterol content increases with postharvest and also with physiological age of the leaves. Sitosterol is the major free sterol present, followed by stigmasterol and campesterol [83].

Emergence has been reported as one negative aspect of the crop, which could be addressed by specific breeding programmes [68]. Chicory can also be propagated and grown by means of micropropagation by regeneration of meristematic nodules. The leaves are cultivated *in vitro* and develop into plantlets when transferred to soil [84]. Although chicory can be regenerated *in vitro* from explants, both through organogenesis and somatic embryogenesis, and from protoplasts, no transgenic plants have yet been reported that have been produced [85]. 

Promising potential utility technologies of the plant have emerged. Chicory roots pulps are an important by-product of the inulin processing industries and are usually used in animal feed. Other applications can be found for these materials since the extraction of chicory pulp yields high levels of pectin, a polysaccharide extensively used in food as a gelling agent, thickening agent, and stabilizer [86]. The crude protein content in chicory is more valuable than in alfalfa. Furthermore the crude lipid in chicory is generally higher than most varieties of alfalfa [9]. It also has a nutritional quality comparable to lucerne, with a mineral content superior for copper and zinc, with similar proportions of protein, lipid, minerals, and other nutrients, and palatable with good digestion with applicability in the poultry and livestock industry [87, 88].

## 8. Conclusion

*Cichorium intybus *has a long tradition of use globally. Historically, chicory was grown by the ancient Egyptians as a medicinal plant, coffee substitute, and vegetable crop and was occasionally used for animal forage. This multipurpose plant contains high amounts of proteins, carbohydrates, and mineral elements [9]. Inulin from chicory roots is considered a functional food ingredient as it affects physiological and biochemical processes resulting in better health and reduction of the risk of many diseases [89].

To date, chicory remains an extremely versatile plant, amenable to genetic manipulation, and there is interest shown in genetically engineered chicory to obtain higher yields and create new potentials [1]. The documented indigenous knowledge relating to the various medicinal uses of chicory has been supported by phytochemical isolation and investigations into biological activity. Nonetheless, many of its constituents have not been explored for their pharmacological potential and further research is necessary to gain better understanding of the phytochemicals against various diseases [9]. Toxicological data on *C. intybus* is currently limited; however, considering that the Asteraceae family is a known source of allergic problems, a contraindication for hypersensitivity should be included in the safety data [5]. Recent studies suggest the use of *C. intybus* as a biomonitor for heavy metals [90, 91]; considering that chicory enters the food chain, this plant should be used with caution. The apparent bioactivity of *C. intybus *shown in preclinical studies (both* in vitro* and* in vivo*) is a testament to its historical use in traditional medicine.

## Figures and Tables

**Table 1:** Traditional medicinal uses of *Cichorium intybus*.

Country	Traditional use(s)	Plant part(s)	Preparation(s)	Reference
Afghanistan	Malaria	Root	Aqueous extract	[12]

Bosnia and Herzegovina	Diarrhea, strengthening the prostate and other reproductive organs, pulmonary cancer, hangover, and purification of biliary tract	Aerial part, flowers, roots	Not stated	[92]
	Liver disorders, spasmolytic, cholesterol, antiseptic	Aerial	Decoction	[15]

Bulgaria	Cholagogue stimulant for gastric secretion, hypoglycemic	Roots, aerial parts	Decoction	[93]

India	Liver disorders	Seeds		[14]
	Diabetes	Whole plant	Not stated	[17]
	Jaundice, liver enlargement, gout, and rheumatism	Root	Decoction	[17]
	Cough relief	Not stated		

Iran	Eupeptic, stomachic, depurative, choleretic, laxative, hypotension, tonic, and antipyretic	Whole plant	Not stated	[94]

Italy	Blood cleansing	Leaves	Not stated	[13]
	High blood pressure	Leaves	Decoction	[95]
	Blood purification, arteriosclerosis, antiarthritis, antispasmodic, digestive	Leaves/roots	Decoction	[96]

	Depurative	Whorls	Decoction	[97]
	Choleretic, hepatoprotective against jaundice, mild laxative, hypoglycemic	Leaves	Decoction, squashed fresh leaves	[93]

Jordan	Internal hemorrhage, sedative in typhoid	Whole plant	Cooking	[14]

Morocco	Renal disease	Aerial/roots	Not stated	[98]
	Kidney disorders, diabetes	Whole plant	Decoction	[99]

Pakistan	Diabetes	Roots	Decoction	[43]

Poland	Digestive complaints and lack of appetite	Roots	Tea	[5]

Serbia	Diarrhea	Flower	Infusion	[100]
	Diuretic, digestive, laxative, anti-inflammatory, liver complaints, reducing blood sugar	Roots	Decoction/tea	[16]
	Cholagogue, digestive, hypoglycemic	Aerial part/root	Not stated	[101]

South Africa	Jaundice, tonic	Leaves, stems, roots		[3]

Turkey	Cancer, kidney stones	Roots	Decoction	[7]
	Wound healing	Leaf	Ointment	[10]
	Hemorrhoids, urinary disorders	Aerial	Tea	[102]

**Table 2:** Compounds isolated and identified from *Cichorium intybus* (chicory).

Compound	Reference(s)
Lactucin	[12, 31, 103]
Lactucopicrin	[12, 31]
8-Deoxylactucin	[31, 104]
Jacquilenin	[103]
11*β*,13-Dihydrolactucin	[103]
11,13-Dihydrolactucopicrin	[103, 104]
Crepidiaside B	[103]
Cyanidin 3-O-p-(6-O-malonyl)-D-glucopyranoside	[105]
3,4*β*-Dihydro-15-dehydrolactucopicrin	[103]
Magnolialide	[103]
Ixerisoside D	[103]
Loliolide	[103]
Cichorioside B	[103, 104]
Sonchuside A	[103, 104]
Artesin	[103]
Cichoriolide	[103]
Cichorioside	[103]
Sonchuside C	[103]
Cichopumilide	[103]
Putrescine	[83]
Spermidine	[83]
*β*-Sitosterol	[7, 83]
Campesterol	[83]
Stigmasterol	[83]
(7S, 8R)-3′-Demethyl-dehydrodiconiferyl alcohol-3′-O-*β*-glucopyranoside	[106]
Chlorogenic acid	[19, 44, 106]
3,5-Dicaffeoylquinic acid	[18, 106]
4,5-Dicaffeoylquinic acid	[106]
Crepidiaside A	[106]
Cichoralexin	[26]
Malic acid	[18]
Caffeic acid	[18, 44]
3-Caffeoylquinic acid	[18]
5-Caffeoylquinic acid	[18]
4-Caffeoylquinic acid	[18]
*cis*-5-Caffeoylquinic acid	[18]
*cis*-Caftaric acid	[18]
*trans*-Caftaric acid	[18]
5-Caffeoylshikimic acid	[18]
5-*p*-Coumaroylquinic acid	[18]
Quercetin-3-O-glucuronide-7-O-(6′′-O-malonyl)-glucoside	[18]
Kaempferol-3-O-glucosyl-7-O-(6′′-O-malonyl)-glucoside	[18]
Dimethoxycinnamoyl shikimic acid	[18]
Kaempferol-3-O-sophoroside	[18]
Isorhamnetin-7-O-(6′′-O-acetyl)-glucoside	[18]
5-O-Feruloylquinic acid	[18]
Dicaffeoyltartaric acid (chicoric acid)	[18]
Kaempferol-7-O-glucosyl-3-O-(6′′-malonyl)-glucoside	[18]
Delphinidin-3-O-(6′′-O-malonyl)-glucoside-5-O-glucoside	[18]
Cyanidin-3,5-di-O-(6′′-O-malonyl)-glucoside	[18]
Cyanidin-3-O-(6′′-O-malonyl)-glucoside	[18]
Petunidin-3-O-(6′′-O-malonyl)-glucoside	[18]
Cyanidin	[18, 105]
Cyanidin-3-O-galactoside	[18]
Cyanidin-3-O-glucoside	[18, 105]
Cyanidin-3-O-(6′′-O-acetyl)-glucoside	[18]
Malvidin-3-O-glucoside	[18]
Pelargonidin-3-O-monoglucuronide	[18]
4-O-Feruloylquinic acid	[18]
Apigenin-7-O-glucoside	[18]
Chrysoeriol-3-O-glucoside	[18]
Tricin-3-O-glucoside	[18]
1,3-Dicaffeoylquinic acid	[18]
1,4-Dicaffeoylquinic acid	[18]
3,4-Dicaffeoylquinic acid	[18]
Quercetin-7-O-galactoside	[18]
Quercetin-3-O-(6′′-O-malonyl)-glucoside	[18]
Quercetin-7-O-glucoside	[18]
Quercetin-7-O-glucuronide	[18]
Quercetin-7-O-(6′′-O-acetyl)-glucoside	[18]
Kaempferide glucuronide	[18]
Kaempferol-7-O-glucoside	[18]
Kaempferol-7-O-rutinoside	[18]
Quercetin-7-O-p-coumaroylglucoside	[18]
Isorhamnetin-7-O-neohesperidoside	[18]
Kaempferol-7-O-(6′′-O-malonyl)-glucoside	[18]
Kaempferol-7-O-glucuronide	[18]
Kaempferide-3-O-(6′′-O-malonyl)-glucoside	[18]
Kaempferol-3-O-glucuronide	[18]
Kaempferol-3-O-glucuronide-7-O-glucoside	[18]
Kaempferol-3-O-(6′′-O-malonyl)-glucoside	[18]
Kaempferol-3-O-glucoside	[18]
Myricetin-7-O-(6′′-O-malonyl)-glucoside	[18]
Kaempferol-7-O-neohesperidoside	[18]
Kaempferol-7-O-(6′′-O-acetyl)-glucoside	[18]
Kaempferol-3-O-(6′′-O-acetyl)-glucoside	[18]
Isorhamnetin-7-O-glucoside	[18]
Isorhamnetin-7-O-glucuronide	[18]
Delphinidin 3,5-di-O-(6-O-malonyl-*β*-D-glucoside)	[19]
Delphinidin 3-O-(6-O-malonyl-*β*-D-glucoside)-5-O-*β*-D-glucoside	[19]
Delphinidin 3-O-*β*-D-glucoside-5-O-(6-O-malonyl-*β*-D-glucoside)	[19]
Delphinidin 3,5-di-O-*β*-D-glucoside	[19]
3-O-p-Coumaroyl quinic acid	[19]
Cyanidin 3-O-*β*-(6-O-malonyl)-D-glucopyranoside	[105]
Quercetin 3-O-*β*-D-glucoside	[19]
Oxalic acid	[67]
Shikimic acid	[67]
Quinic acid	[67]
Succinic acid	[67]

**Table 3:** The volatile constituents of *Cichorium intybus* (adapted from Judžentienė and Būdienė [4]).

Compound
Octane
Octen-3-ol
2-Pentyl furan
(2*E*, 4*E*)-Heptadienal
1,8-Cineole
Benzene acetaldehyde
*n*-Nonanal
Camphor
(2*E*, 6Z)-Nonadienal
(2*E*)-Nonen-1-al
*n*-Decanal
(2*E*, 4*E*)-Nonadienal
*n*-Decanol
(2*E*, 4Z)-Decadienal
*n*-Tridecane
(2*E*, 4*E*)-Decadienal
*β*-Elemene
(*E*)-Caryophyllene
*β*-Ylangene
Geranyl acetone
(*E*)-*β*-Farnesene
*allo*-Aromadendrene
dehydro-Aromadendrene
*β*-Ionone
Pentadecane
*trans*-*β*-Guaiene
(2*E*)-Undecanol acetate
Sesquicineole
(2*E*)-Tridecanol
*n*-Hexadecane
Tetradecanal
Tetradecanol
2-Pentadecanone
(*E*)-2-Hexylcinnamaldehyde
Octadecane
*n*-Nonadecane
(5*E*, 9*E*)-Farnesyl acetone
*n*-Eicosane
*n*-Octadecanol
*n*-Heneicosane
